# Characterization of Chikungunya Virus-Like Particles

**DOI:** 10.1371/journal.pone.0108169

**Published:** 2014-09-29

**Authors:** Nitchakarn Noranate, Naokazu Takeda, Prukswan Chetanachan, Pathompong Sittisaman, Atchareeya A-nuegoonpipat, Surapee Anantapreecha

**Affiliations:** 1 Thailand-Japan Research Collaboration Center on Emerging and Re-emerging Infections (RCC-ERI), Nonthaburi, Thailand; 2 National Institute of Health, Department of Medical Sciences, Ministry of Public Health, Nonthaburi, Thailand; 3 Research Institute for Microbial Diseases, Osaka University, Osaka, Japan; University of Pittsburgh, United States of America

## Abstract

Chikungunya virus (CHIKV) is becoming a global concern due to the increasing number of outbreaks throughout the world and the absence of any CHIKV-specific vaccine or treatment. Virus-like particles (VLPs) are multistructured proteins that mimic the organization and conformation of native viruses but lack the viral genome. They are noninfectious and potentially safer vaccine candidates. Recent studies demonstrated that the yield of CHIKV VLPs varies depending on the strains, despite the 95% amino acid similarity of the strains. This might be due to the codon usage, since protein expression is differently controlled by different organisms. We optimized the region encoding CHIKV structural proteins, C-E3-E2-6k-E1, inserted it into a mammalian expression vector, and used the resulting construct to transfect 293 cells. We detected 50-kDa proteins corresponding to E1 and/or E2 in the cell lysate and the supernatant. Transmission electron microscopy revealed spherical particles with a 50- to 60-nm diameter in the supernatant that resembled the native CHIKV virions. The buoyant density of the VLPs was 1.23 g/mL, and the yield was 20 µg purified VLPs per 10^8^ cells. The VLPs aggregated when mixed with convalescent sera from chikungunya patients, indicating that their antigenicity is similar to that of native CHIKV. Antibodies elicited with the VLPs were capable of detecting native CHIKV, demonstrating that the VLPs retain immunogenicity similar to that of the native virion. These results indicated that CHIKV VLPs are morphologically, antigenically, and immunologically similar to the native CHIKV, suggesting that they have potential for use in chikungunya vaccines.

## Introduction

Chikungunya fever is a mosquito-borne disease in Africa, South Asia, and Southeast Asia that usually starts 2–4 days after chikungunya virus (CHIKV) infection. The clinical symptoms include high fever, rash, headache, vomiting, myalgia, and joint pain [1]. Because a mosquito vector, *Aedes albopictus*, is present in habitats across Europe, North America, and East Asia, CHIKV infections have become a serious public health concern [2]–[6]. The most recent outbreaks began in Kenya in 2004, and CHIKV has subsequently been introduced into many countries [7]–[9]. Although CHIKV was first isolated in Tanzania in 1953 and the recent epidemics have continued to underscore the need for therapeutic and preventive measures, there are still no treatment agents or vaccines for this infection [10].

CHIKV is a member of the genus *Alphavirus* in the family *Togaviridae*. The genome of CHIKV is composed of a positive-sense single-stranded RNA genome of 11.7 kb encoding four non-structural and five structural proteins. The structural proteins are translated from a subgenomic 26S mRNA as a single polyprotein, which is processed cotranslationally into five structural proteins: capsid, E3, E2, 6K and E1 [11].

Virus-like particles (VLPs) are multiprotein structures that mimic the organization and conformation of the authentic native virus, but lack the viral genome [12] and are therefore noninfectious. Since VLPs are morphologically similar to the native virion, they could be a better candidate for a safe vaccine. Using experimentally generated VLPs, Brady (1978) demonstrated that viral capsid proteins self-assemble into VLPs in the absence of viral genetic material [13]. VLP-based vaccines are currently available for hepatitis B and cervical cancer, and Norwalk virus and influenza virus VLPs are undergoing clinical trials [12]. In addition, several preclinical trials using VLPs from reovirus, hepatitis C virus, and lentivirus are ongoing [14].

It was shown in 2010 that the expression of CHIKV structural polyproteins in culture cells resulted in the formation of VLPs which showed high immunogenicity and induced a protective immune response in non-human primates [15]. The production levels for the individual VLPs differed among CHIKV isolates, despite the high similarity of their coding sequences [15]. Although key amino acids that regulate particle formation and production have been revealed, the protein expression is differently controlled among organisms [16]. The quantity of the heterologously expressed proteins varies greatly among cell types, because mammalian cells show a complicated process of post-translational modifications, and because the level of protein production is affected by codon usage, as shown previously in non-permissive cells [17].

In the present study we characterized CHIKV VLPs containing optimized codons generated in a human-derived cell line.

## Materials and Methods

### Ethics statement

All animal experiments were reviewed and carried out according to “Guides for animal experiments performed at the Department of Medical Sciences, Ministry of Public Health, Thailand”. The protocol was approved by the committee on the ethics of Animal Experiments of the Department of Medical Sciences, Ministry of Public Health under the permission number 55-012. After experiments were performed, all animals were euthanized by anesthesia with CO_2_ and collected whole blood from heart.

### Cells, viruses and antisera

293T cells were grown in Dulbecco's Modified Eagle's medium (DMEM) containing 10% fetal bovine serum. 293F cells were maintained with Freestyle 293 expression medium (Gibco, Carlsbad, CA). The CHIKV 32808 strain was isolated in Thailand in 2008, and the 14635 and 16856 strains were isolated in Thailand in 2009. The Ross strain was obtained from the National Institute of Health (NIH), Thailand.

The CHIKV strains were purified by three successive rounds of plaque cloning in Vero cells and stocked in a small aliquot at −80°C. The convalescent sera 32095, 32097, and 32221, were from three chikungunya patients infected during the 2010 epidemic in Thailand.

### Construction of expression plasmids

**Figure 1:**
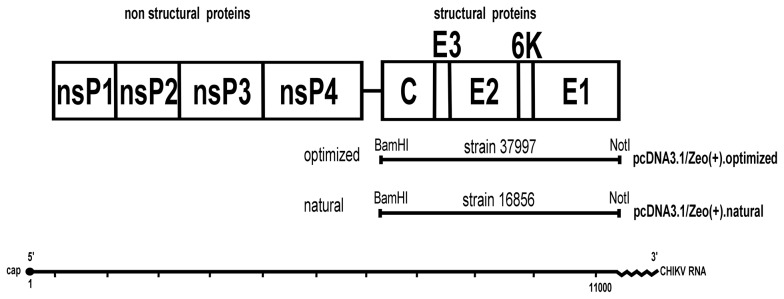
Genome organization of CHIKV and an expression vector. A synthetic DNA fragment encoding human-preferred CHIKV structural protein genes, C-E3-E2-k6-E1, was subcloned into the plasmid pcDNA3.1/zeo^+^ to generate the expression vector. A DNA fragment encoding natural CHIKV structural protein genes was amplified and subcloned into the same vector.

The CHIKV structural protein genes, capsid-E3-E2-6K-E1, encoding 3747 bp were obtained from two different sources. One was the synthetic genes consisting of human-preferred codons based on the amino acid sequence of CHIKV strain 37997 obtained from GenBank (accession number EU224270), which were inserted into a plasmid pUC57 (GenScript, Piscataway, NJ) as described [18]. The optimized fragment was subcloned into an expression vector, pcDNA3.1/zeo^+^ (Invitrogen, Carlsbad, CA), using BamHI and NotI sites to construct pcDNA3.1/zeo(+).optimized. The other source was a CHIKV strain isolated from a Thai patient in 2009. A forward primer containing a BamHI site (underlined), 5′-ATATTTGGATCCAATGGAGTTCATCCCAACCCAAACT-3′, and a reverse primer containing a NotI site (underlined), 5′-TATATTGCGGCCGCTTAGTGCCTGCTGAACGACACGCA-3′ were used to amplify a fragment encoding the structural proteins, which was cloned into pCR-XL-TOPO (Invitrogen) and subcloned into the expression vector, pCDNA3.1/zeo^+^, using the same strategy described above to construct pcDNA3.1/zeo(+).natural. Both the optimized and natural sequences were verified by sequencing (ABI 3100, Applied Biosystems, Foster City, CA) (Fig. 1).

### Expression of CHIKV structural proteins and purification of VLPs

We transfected 293T cells with 4 µg/5×10^5^ cells of the plasmid by using the Lipofectamine 2000 method as specified by the manufacturer (Invitrogen). The time-course experiments regarding the expression of VLPs were performed with 293T cells in T25 flasks (Corning, Tewksbury, MA). The VLPs were recovered by centrifugation at 100,000×g for 2 h at 4°C in a SW55 Ti rotor (Beckman Coulter, Indianapolis, IN), resuspended in 50 µL of TNE buffer (0.01 M Tris-HCl, pH 7.2, 0.1 M NaCl, 0.001 M EDTA), and kept at 4°C. Next, 20 µL of the suspension was loaded onto 4%–12.5% sodium dodecyl sulfate polyacrylamide gel electrophoresis (SDS-PAGE), and the separated proteins were transferred onto a PVDF membrane (GE Healthcare, Piscataway, NJ) using a semidry system (Bio-Rad, Hercules, CA).

The membrane was blocked with 5% skim milk (BD Biosciences, Franklin Lakes, NJ) in phosphate-buffered saline PBS (Sigma, St. Louis, MO) containing 0.1% Tween-20 (PBST) for 1 h at room temperature (RT) or overnight at 4°C. The membrane was then washed with PBST and subsequently incubated for 1 h at RT with chikungunya patient serum (1∶3000 dilution) or CHIKV-immunized mouse sera (1∶3000 dilution) with PBST containing 5% skim milk. The membranes were then washed three times with PBST and treated with PBST containing horseradish peroxidase (HRP)-conjugated rabbit anti-human serum (1∶5,000 dilution) or goat anti-mouse serum (Dako, Glostrup, Denmark) (1∶5,000 dilution) for 1 h at RT. After three washes with PBST, the proteins were detected using ECL prime reagents and a Hyperfilm ECL system (GE Healthcare) according to the manufacturer's instructions.

The VLPs were also generated by transfecting 293F cells with the expression vectors using cationic lipid, 293fectin (Invitrogen), according to the manufacturer's instructions. The supernatant was harvested at 72 h post-transfection (p.t.), centrifuged at 2,000 × g for 10 min to remove the cells and debris, and further clarified by centrifugation at 10,000 × g for 20 min at 4°C. The VLPs in the supernatant were concentrated by ultracentrifugation through a 15% sucrose cushion at 100,000 × g for 2 h at 4°C.

The pellet was gently resuspended in TNE buffer, and the VLPs were further purified by CsCl density gradient equilibrium centrifugation at 100,000 × g for 16 h at 4°C in an SW55 Ti rotor. Ten fractions were collected from each centrifugation tube and the specific gravity was measured. The CHIKV structural proteins were then detected by a western blot analysis. The fractions containing the structural proteins were pooled, diluted in TNE buffer, and centrifuged at 100,000×g for 2 h at 4°C to remove CsCl. The concentration of the purified VLPs was determined by the Bradford method as outlined by the manufacturer (Bio-Rad).

### Transmission electron microscopy (TEM)

The purified VLPs were coated onto formvar carbon film of 400-mesh copper grids for 1 min at RT, gently air-dried, and stained with 4% uranyl acetate for 30 s. The samples were examined under a transmission electron microscope (TEM; model JEM-1010, JEOL, Tokyo) working at 75kV.

### Immunoelectron microscopy (IEM)

The purified VLPs (15 ng in 10 µL) derived from the optimized sequence were incubated with an equal volume of diluted convalescent human serum (1∶150), undiluted normal human serum, or TNE buffer for 1 h at 37°C. The antigen-antibody complex was stained with uranyl acetate and examined directly by TEM as described above.

### ELISA to detect antibodies against CHIKV

We measured the antibody titers against CHIKV by using an enzyme-linked immunosorbent assay (ELISA). Briefly, 100 µL/well of purified VLPs (50 ng/mL) was coated onto a 96-well plate (Thermo Scientific, Waltham, MA) and incubated overnight at 4°C. The wells were blocked with 200 µL/well of 5% skim milk in PBST and incubated at 37°C for 1 h. After four washes with PBST, 100 µL/well of serially diluted sample serum was added and the plate was incubated at 37°C for 1 h. After the wells were washed again four times with PBST, 100 µL of HRP-conjugated antibody diluted with 1% PBST (1∶16,000) was added and the plate was incubated at 37°C for 1 h.

After the plate was washed with 200 µL/well of PBST four times, SureBlue Reserve TMB Microwell Peroxidase 1-Component (KPL, Gaithersburg, MD) was added at 100 µL/well and incubated at RT for 30 min in the dark. Stop solution, 0.6N H_2_SO_4_, was added at 100 µL/well and the optical density (O.D.) was immediately measured at 450 nm and also at 620 nm for background subtraction by using a Multiskan FC Microplate Photometer (Thermo Scientific). The experiments were performed twice in duplicate.

### ELISA to detect CHIKV antigens

Hyperimmune sera against CHIKV 16856 strain and VLPs were prepared with rabbits and guinea pigs. A rabbit and a guinea pig were immunized subcutaneously with 25 µg of the purified CHIKV or VLPs with Freund's complete adjuvant. After 4 weeks, the animals received one booster injection of the same dose of CHIKV or VLPs with Freund's incomplete adjuvant. The animals were bled one week after the booster injection. Mouse sera were prepared as described [18].

We conducted an ELISA to detect CHIKV antigen. Hyperimmune serum from the rabbit prepared as described above was used to capture CHIKV antigen, and hyperimmune serum from the guinea pig was used as a detector antibody. A 96-well plate (Thermo Scientific) coated with 100 µL/well of hyperimmune or preimmune rabbit serum (1∶10,000) was incubated overnight at 4°C, and the wells were blocked with 5% skim milk in PBST and incubated at 37°C for 2 h. After the blocking solution was discarded, the plate was washed twice with PBST (200 µL/well). Serially diluted CHIKV antigen was added and the plate was incubated at 4°C overnight. After the wells were washed three times with PBST, 100 µL/well of guinea pig hyperimmune serum (1∶1,000) was added and the plate was incubated at 37°C for 2 h. After the wells were washed four times with PBST (200 µL/well), 100 µL of HRP-conjugated anti-guinea pig serum (1∶3,000) per well was added and the plate was incubated at 37°C for 1 h. After the wells were washed five times with PBST, 100 µL/well of SureBlue Reserve TMB Microwell Peroxidase 1-Component (KPL) was added and the plate was incubated at RT for 30 min in the dark. Stop solution, 100 µL/well of 0.6N H_2_SO_4_, was added and the O.D. was immediately measured at 450 nm and also at 620 nm for background subtraction by using a Multiskan FC Microplate Photometer (Thermo Scientific). The experiments were performed in duplicate.

### Microneutralization test

The neutralization test was performed with a 96-well microplate. The serum samples (50 µL) were serially diluted twofold starting at a dilution of 1∶10. The dilutions were mixed with an equal volume of CHIKV Ross strain containing 100 TCID_50_/50 µL, and incubated for 1 h at 37°C in a 5% CO_2_ incubator. Vero cells (10^4^ cells/100 µL) were added to each dilution and incubated at 37°C in a 5% CO_2_ incubator. We determined the cell viability at 48 h by using WST1 in a cell proliferation assay (Roche, Upper Bavaria, Germany) according to the manufacturer's instructions. The neutralizing antibody titer was calculated from the highest serum dilution that protected ≥ 50% cell viability compared with the virus control. All samples were tested in duplicate.

### Stable cell line for VLPs production

One day prior to transfection, we seeded the 293T cells into a 24-well plate at a density of 6×10^4^ cells/cm^3^. The cells were transfected with the VLP expression plasmid using the Lipofectamine 2000 method as specified by the manufacturer (Invitrogen). Two days after transfection, the cells were removed from the wells and diluted with culture medium (1∶10 ratio), subjected to limiting dilution (twofold dilution starting at 1∶10 to 1∶1280, eight dilutions in total) and transferred to a 12-well plate.

After 24 h of incubation, the medium was replaced with fresh medium containing 200–400 µg/mL Zeocin (Invitrogen) to select resistant cells. We replaced the selective medium every 3 days until Zeocin-resistant cells were formed. When the cells became confluent, they were transferred to larger vessels. The culture supernatants were collected and examined for VLPs as described above. The expression of the structural proteins was examined by an immunofluorescence assay.

### Immunofluorescence assay (IFA)

We seeded the cells onto 8-well chamber slides (Iwaki, Newport, UK) in the selective medium and incubated them at 37°C in a 5% CO_2_ incubator. After 3 days, the selective medium was removed and confluent monolayer cells were washed with PBS and air dried. The cells were fixed with 4% paraformaldehyde in PBS at RT for 20 min. The cell membrane was permeabilized for 5 min at RT with 0.1% Triton X in PBS, washed three times with PBS and then incubated with blocking solution (1% bovine serum albumin [BSA] in PBS) for 1 h. The cells were incubated in a moist chamber with a 1∶2,000 dilution of the CHIKV-immunized guinea pig sera at 37°C for 30 min, washed three times with PBS, and incubated with a 1∶2,000 dilution of the anti-guinea pig antibody-conjugated AlexaFluor 488 (Invitrogen). The sample was examined under a Biozero fluorescence microscope (Keyence, Osaka, Japan).

## Results

### Expression of structural proteins in mammalian cells

We constructed the plasmid expressing structural polyproteins, C-E3-E2-6K-E1, derived from either optimized or natural codons, and used them to transfect mammalian 293T cells (Fig. 1). The supernatant was harvested at 72 h p.t., and the proteins were analyzed by western blot analysis using antiserum from a mouse immunized with formalin-inactivated CHIKV. The predicted molecular masses of the CHIKV structural proteins were 138 kDa for C-E3-E2-6K-E1, 62 kDa for P62 (E3/E2), 47 kDa for both E2 and E1, 29 kDa for capsid, and 6 kDa for 6k.

**Figure 2:**
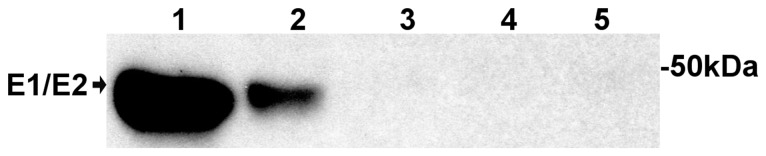
Western blot assay of structural proteins expressed in 293T cells. We transfected 293T cells with an expression plasmid containing structural proteins derived from either natural or optimized codons. The supernatant was analyzed by western blot analysis using serum from a mouse immunized with formalin-inactivated CHIKV. Results for the following are shown: (1) the supernatants from 293T cells transfected with optimized structural protein genes, (2) natural structural protein genes, (3) plasmid vector, (4) no plasmid vector but lipofectamine, and (5) no transfection.

As shown in Figure 2, immunoreactive protein bands with molecular masses of approx. 47 kDa were detected in the supernatants from the cells transfected with both optimized and natural codon-based plasmids. This size corresponds to the predicted molecular mass of the envelope glycoprotein E2 and/or E1, whereas no immunoreactive band was detected when pooled serum obtained from healthy individuals was used (data not shown). These results indicated that the structural proteins were produced and released into the supernatant. It is noteworthy that the expression of the proteins with the optimized codons was much higher than that obtained with the natural codons.

### Formation of CHIKV VLPs

**Figure 3:**
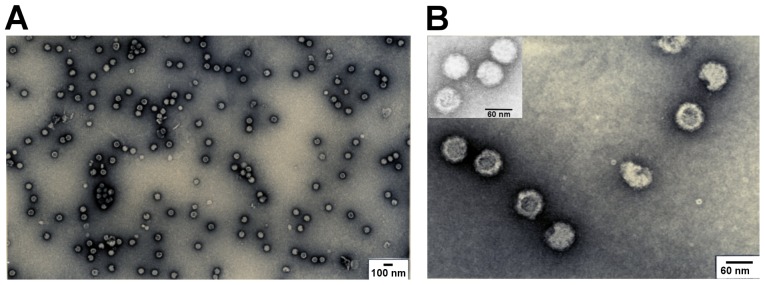
TEM analysis of purified VLPs expressed with the optimized codons. (A) Magnification at ×30,000, (B) ×150,000, including CHIKV virion (top, left corner).

To examine whether the expressed structural proteins self-assemble into VLPs, we subjected the supernatant from 293F cells transfected with the optimized codons and recovered at 72 h p.t. to ultracentrifugation as described in the Materials and Methods. The final pellet was resuspended and observed with TEM. We found many regular spherical particles with external diameters of 50–60 nm (Fig. 3A, and 3B). The morphology of the particles resembled authentic CHIKV virions.

**Figure 4:**
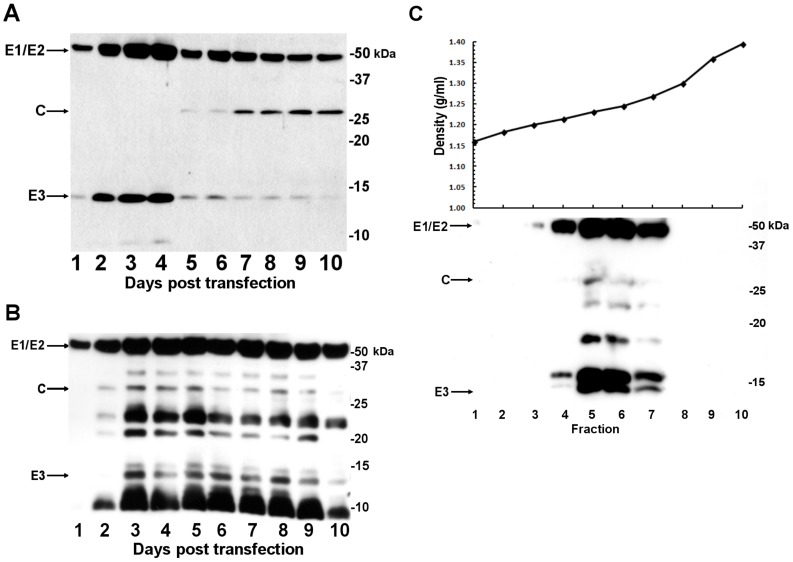
Time course of the formation of CHIKV VLPs. 293T cells were transfected with the expression plasmid, and the supernatant was collected for 10 consecutive days. The supernatant (20 µL) was subjected to the VLP concentration as described in the Materials and Methods, and analyzed by western blotting using anti-CHIKV mouse serum. (A) Time course of the expression by the plasmid with the natural codons. (B) Results for the same experiment but using the plasmid with optimized codons. Lanes 1 through 10 are days 1 to 10 p.t. (C) CsCl equilibrium density gradient centrifugation of the VLPs purified from 293F cells. The proteins in each fraction were analyzed by western blotting.

To monitor the VLP formation and its yield, we transfected and incubated 293T cells for 10 days. During this time, the supernatant was harvested every 24 h, and the VLPs were purified and examined by western blot analysis (Fig. 4). Major proteins of approx. 50 kDa which corresponded to the envelope glycoproteins E1 and/or E2 were found at day 1 p.t. in the cells transfected with both natural and optimized codons. The expression level of 50-kDa proteins with the natural codons reached a maximum at day 4 p.t. and then declined gradually over the next 6 days (Fig. 4A), whereas that with the optimized codons reached a maximum at days 3 to 4 p.t and remained an additional 7 days p.t. (Fig. 4B).

In addition, a protein band with a molecular mass of 130 kDa was observed between days 3 to 9 p.t. (data not shown). The size of the 130-kDa protein was in agreement with the predicted primary translation product of the structural polyprotein. This expressed protein was not observed in the cells expressing proteins with the natural codons (data not shown). These results again demonstrated that a higher formation and yield of the VLPs occurred in the cells expressing the proteins with the optimized codons compared to those with the natural codons. The buoyant density of the VLPs was 1.23 g/mL in CsCl (Fig. 4C). This value is similar to the 1.24 g/mL observed in the native virus particles, although 1.23 g/mL was reported in another study [19].

We performed a large-scale expression of the structural proteins using 293F cells as described in the Materials and Methods. The purity of VLP was measured by SDS-PAGE followed by Coomassie blue staining as well as a western blot analysis with a rabbit antibody elicited against the purified CHIKV, and no protein band other than viral capsid proteins was detected. The yield of purified VLPs was approx. 20 µg per 10^8^ 293F cells.

### Antigenicity of VLPs

**Figure 5:**
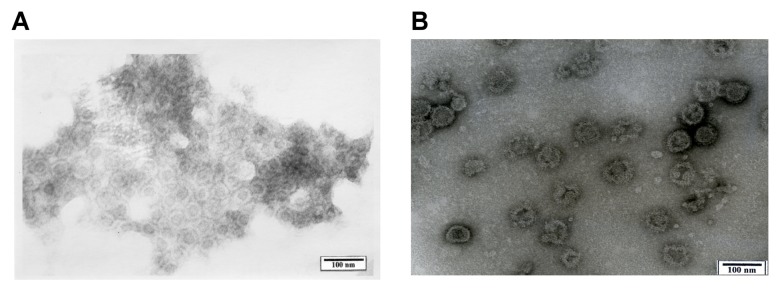
The antigen-coated aggregation of the VLPs observed by immunoelectron microscopy. The antigen-coated aggregation of the VLPs was observed by TEM. The VLPs incubated with (A) serum from a patient (32097), and (B) with serum from a healthy individual.

To examine the antigenicity of the VLPs, we incubated the purified VLPs with a chikungunya patient's serum, and the resultant antigen-antibody complexes were examined using TEM. As shown in Figure 5, the VLPs were coated with the antibodies, resulting in massive aggregations (Fig. 5A), whereas no aggregation was observed when the VLPs were incubated with normal human serum (Fig. 5B), indicating that the VLPs had antigenicity similar to native CHIKV.

**Figure 6:**
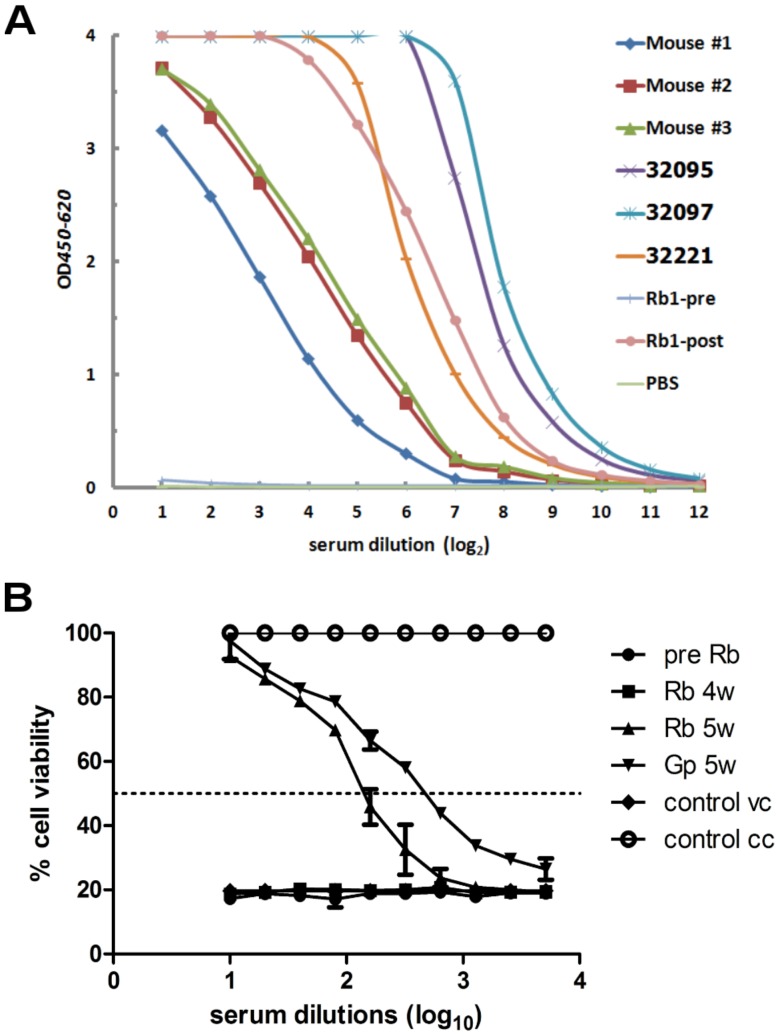
Antigenicity and immunogenicity of VLPs. (A) Anti-CHIKV IgG responses. Microplates were coated with purified VLPs (5 ng/well) and used to detect CHIKV-specific antibodies. IgG antibodies in the 1∶50 diluted serum from one rabbit (Rb1-post) and the sera from three mice (mouse #1, #2 and #3) immunized with inactivated CHIKV, and three sera from convalescent chikungunya patients (# 32095, 32097 and 32221) were examined. Preimmune (Rb1-pre) and PBS were included as controls. (B) Microneutralizaton assays to examine the neutralizing activity of anti-VLP sera. Serial twofold dilutions of the preimmune and postimmune sera were mixed with an equal volume of 100 TCID_50_ of the ROSS strain and used to inoculate 10^4^ Vero cells. Cell viability was measured at 48 h after incubation by using a WST1 assay according to the manufacturer's instructions. The serum dilution at 50% cell viability indicated by a broken line was defined as the neutralizing antibody titer. pre Rb; rabbit preimmune serum, Rb 4w; rabbit serum from 4 wks postimmunization, Rb 5w; rabbit serum from 5 wks postimmunization, Gp 5w; guinea pigs serum from 5 wks postimmunization, vc; virus control, cc; cell control.

To further examine the antigenic specificity, we used the VLPs as the antigen to coat microplates and we then examined the reactivity to CHIKV-specific antibodies. Twofold serial dilutions of the serum from a rabbit serum, sera from three mice immunized with inactivated CHIKV, and the sera from three convalescent patients with CHIKV were subjected to the ELISA to detect antibodies. As depicted in Figure 6A, increasing reactivity was observed in the rabbit serum sample (ELISA titer of 1∶204,800 for Rb1-post) and three mouse sera samples (ELISA titers of 1∶51,200, 1∶204,800 and 1∶102,400 for mouse #1–3, respectively). Similar reactivity was observed when the three patients' sera (ELISA titers of 1∶204,800 for 32095, 1∶204,800 for 32097 and 1∶102,400 for 32221) were examined, demonstrating that the VLPs possess antigenicity similar to that of native CHIKV and are capable of serving as an antigen to detect CHIKV-specific antibodies.

### Immunogenicity of VLPs

Hyperimmune sera against VLPs were prepared with rabbits and guinea pigs as described in the Materials and Methods. The preimmune and high titer rabbit sera were used to coat the microplate and then incubated with the culture fluid from CHIKV-infected 293T cells. The reactivity of the supernatant from the mock-infected cells was less than 1∶20. Preimmune sera from the rabbit were not able to capture CHIKV antigen. The ELISA titers of the culture medium were 1∶320 for the CHIKV ROSS strain and 1∶640 for the CHIKV 16856 strain. These results demonstrated that the VLPs were immunogenic and were able to elicit antibodies capable of binding to native CHIKV antigen.

To examine the neutralizing activity of VLP-immunized rabbit and guinea pig sera, a viability assay was performed using the CHIKV Ross strain as the challenge virus. As depicted in Figure 6B, the titers were 1∶80 and 1∶320 for the rabbit and guinea pig, respectively, indicating that the VLPs were capable of eliciting neutralizing antibodies.

### A stable cell line expressing VLPs

**Figure 7:**
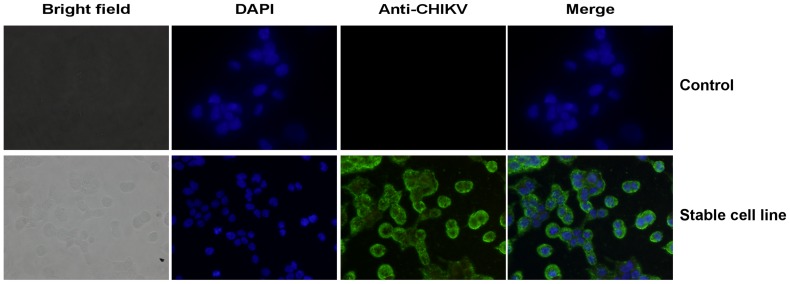
Immunodetection of CHIKV antigens in the stable 293T cell line expressing VLPs. The Zeocin-resistant cell lines were collected and immunostaining was performed with anti-CHIKV serum from a guinea pig. Untransfected 293F cells were used as a negative control. The cells were monitored under a fluorescence microscope.

To establish stable cell lines, we subjected the transfected 293T cells to 10-fold dilutions and incubated them in the presence of Zeocin. After 28 days of incubation, Zeocin-resistant cells reached confluence. The cells were collected and the expression of the CHIKV structural proteins was examined by IFA. As shown in Figure 7, CHIKV antigens were mainly detected on the cell surface. The yield of the VLPs in the supernatant derived from one of the stable cell lines was 10 µg per 1.2× 10^8^ cells measured by the Bradford method.

## Discussion

The recent outbreaks of chikungunya fever across several continents highlight the urgency of developing prevention and control methods for this agent [20]. Although many approaches for the development of chikungunya vaccine have been reported such as the use of live attenuated vaccine [21], [22], formalin-inactivated vaccine [23], a recombinant live attenuated vaccine [24], a chimeric alphavirus vaccine [25], DNA vaccines [26], [27] and VLP-based vaccines [15], [28], there is still no licensed vaccine available to prevent CHIKV infection. An attenuated virus vaccine was shown to elicit an immune response, but it was withdrawn during phase II clinical trials [29].

In the present study we expressed CHIKV structural genes, C-E3-E2-6k-E1, which were optimized to the human preferred codons as described [15]. The expressed proteins were self-assembled into VLPs and released into the culture supernatant, and our TEM observations indicated that the VLPs were morphologically similar to the native CHIKV particles. The yield of the VLPs greatly exceeded that of VLPs derived from viral natural codons. This result supported the results of a previous study in that the yield of VLP was different despite the high degree of amino acid similarity [15].

Although we used the same amino acid sequences, the yield of our VLPs (i.e., 20 µg purified VLPs per 10^8^ cells by the transient expression and 10 µg purified VLPs per 10^8^ cells by the stable expression) seems to be lower than the yield of 10–20 mg per liter obtained in the previous study [15]. It was also reported that amino acids located in the acid-sensitive region (ASR) in E2 substantially affect VLP production [16]. However, this is unlikely because the key amino acids in the E2 ASR by which the conformation of E1/E2 stability may be affected were unchanged in the present study. Alphavirus assembly and budding efficiency are affected by several factors, such as palmitoylation of the carboxy terminal of E1 and E2 [30], interaction between E1 and E2 or between the cytoplasmic domain of E2 and capsid proteins [31]–[34], the requirement for cholesterol in the cell membrane [35]–[37], and the pH of endosomes [38]–[40]. In addition to these factors, the codon usage might greatly influence the magnitude of VLP generation.

The importance of neutralizing antibody in controlling chikungunya virus infections has been demonstrated [41]–[43]. In the present study, the VLPs indicated a considerably good immunogenicity in experimental animals. A rabbit and a guinea pig that received a single shot and one booster (5 weeks) generated a high titer of anti-CHIKV antibody that was comparable to that using formalin-inactivated CHIKV [23], suggesting that the VLPs have potential for use in a chikungunya vaccine. A recent study demonstrated that compared to the envelope glycoprotein, CHIKV VLPs provide a superior immune response and protection against CHIKV-induced disease in mice [44], suggesting that VLPs could be an attractive platform for chikungunya vaccine production. The stable cell lines have an advantage because the transfection step, which is tedious and requires expensive reagents, is not needed.

The CHIKV VLPs expressed with mammalian cells [15] and baculovirus/insect cells [28] have been shown to elicit neutralizing antibodies that protect experimental animals from CHIKV challenge. However, the VLPs produced using insect cells showed better immunogenicity than those produced in mammalian cells, and this was likely due to the difference in glycosylation between these cells [28]. VLPs produced in different expression systems are often contaminated with residual host cell components that may stimulate the innate immunity and augment the adaptive immune response or can result in induce allergic reactions and other immunopathological effects [45]. In addition, CHIKV infection has been shown to induce IgG2c responses in contrast to the balanced IgG1/IgG2c response by insect cell-derived VLPs [46]. The difference in immune responses between insect cell-producing VLPs and mammalian cell-producing VLPs as well as the mechanism underlying this difference need to be clarified for safer human vaccine production.
